# Supplementary material for: Impact of KRAS mutations and co-mutations on clinical outcomes in pancreatic ductal adenocarcinoma
Source: NPJ Precis Oncol. 2024 Feb 3;8:27. doi: 10.1038/s41698-024-00505-0 (PMC10838312; doi:10.1038/s41698-024-00505-0)
Supplement: Supplementary file 1 — REPORTING SUMMARY [file 41698_2024_505_MOESM1_ESM.pdf]

nature portfolio

Corresponding author(s): Dan Zhao, MD, PhD

Last updated by author(s): 12/06/2023

Reporting Summary

Nature Portfolio wishes to improve the reproducibility of the work that we publish. This form provides structure for consistency and transparency in reporting. For further information on Nature Portfolio policies, see our Editorial Policies and the Editorial Policy Checklist.

Statistics

For all statistical analyses, confirm that the following items are present in the figure legend, table legend, main text, or Methods section.

n/a	Confirmed	
☐	☒	The exact sample size (*n*) for each experimental group/condition, given as a discrete number and unit of measurement
☐	☒	A statement on whether measurements were taken from distinct samples or whether the same sample was measured repeatedly
☐	☒	The statistical test(s) used AND whether they are one- or two-sided<br>*Only common tests should be described solely by name; describe more complex techniques in the Methods section.*
☐	☒	A description of all covariates tested
☐	☒	A description of any assumptions or corrections, such as tests of normality and adjustment for multiple comparisons
☐	☒	A full description of the statistical parameters including central tendency (e.g. means) or other basic estimates (e.g. regression coefficient) AND variation (e.g. standard deviation) or associated estimates of uncertainty (e.g. confidence intervals)
☐	☒	For null hypothesis testing, the test statistic (e.g. *F*, *t*, *r*) with confidence intervals, effect sizes, degrees of freedom and *P* value noted<br>*Give P values as exact values whenever suitable.*
☒	☐	For Bayesian analysis, information on the choice of priors and Markov chain Monte Carlo settings
☒	☐	For hierarchical and complex designs, identification of the appropriate level for tests and full reporting of outcomes
☒	☐	Estimates of effect sizes (e.g. Cohen's *d*, Pearson's *r*), indicating how they were calculated

Our web collection on statistics for biologists contains articles on many of the points above.

Software and code

Policy information about availability of computer code

Data collection	The MD Anderson Cancer Center Institutional Review Board (IRB) approved the collection of demographics, clinical, and pathological information under IRB protocol 09-0373 and 2023-0091. Informed consent was waived, as per the IRB guidelines for retrospective studies of previously collected clinical and molecular information. The Palantir Foundry software system (Palantir, Denver, CO) was used to query the MD Anderson EHR to identify patients with a confirmed diagnosis of PDAC who underwent somatic tumor tissue mutation testing at MD Anderson from 3/14/1997 to 4/27/2023 for inclusion in the study.<br>Patient demographics, histopathology, tumor grade, surgical history, and mutational profiles were collected from the MD Anderson EHR and tumor registry data using the Foundry system. Histologic classification and grade were collected from the patients' pathology reports. Molecular testing was performed at MD Anderson's molecular diagnostics laboratory, which is College of American Pathologists (CAP) accredited and Clinical Laboratory Improvement Amendments (CLIA) certified. The gene panels used evolved during the study inclusion period, with expanding lists of genes over time. The information on tumor genomic alterations (GAs) was extracted from the available clinical and molecular data. Deidentified information was used for analysis.<br>For the validation PANCAN dataset, PanCAN, in partnership with Tempus (Tempus Labs Inc., Chicago, IL), offers the Know Your Tumor® (KYT) precision medicine service to patients with pancreatic cancer. KYT data is available through the PanCAN SPARK platform (www.pancan.org/spark). Tempus processes, sequences and conducts group-level bioinformatics analyses on tumor biopsy samples. Data is derived from the Tempus xT NGS panel that covers 648 genes with actionable oncologic mutations.
Data analysis	Rstudio 2020 (RStudio, PBC. Boston, MA) were used for the statistical analyses and data visualization. The Oncoplot function within MAFtools was used to visualize the somatic mutation distribution.

For manuscripts utilizing custom algorithms or software that are central to the research but not yet described in published literature, software must be made available to editors and reviewers. We strongly encourage code deposition in a community repository (e.g. GitHub). See the Nature Portfolio guidelines for submitting code & software for further information.

Data

Policy information about availability of data

All manuscripts must include a data availability statement. This statement should provide the following information, where applicable:

- Accession codes, unique identifiers, or web links for publicly available datasets
- A description of any restrictions on data availability
- For clinical datasets or third party data, please ensure that the statement adheres to our policy

The data generated in this study is available upon request from the corresponding author. PanCAN KYT data is available for access upon filling out a data sharing agreement on the following domain: pancan.org/spark.

Research involving human participants, their data, or biological material

Policy information about studies with human participants or human data. See also policy information about sex, gender (identity/presentation), and sexual orientation and race, ethnicity and racism.

Reporting on sex and gender	Sex and gender were not considered in study design.
Reporting on race, ethnicity, or other socially relevant groupings	Race, ethnicity, or other socially relevant groupings were not considered in study design.
Population characteristics	The Palantir Foundry software system (Palantir, Denver, CO) was used to query the MD Anderson EHR to identify patients with a confirmed diagnosis of PDAC who underwent somatic tumor tissue mutation testing at MD Anderson from 3/14/1997 to 4/27/2023 for inclusion in the study. A total of 803 patients with PDAC who had tumor tissue somatic mutation testing at MD Anderson were identified; the demographic and clinical characteristics of this cohort are summarized in Table 1. The median age was 63 years (range 26-86), 43% were female, and 29.3% had a smoking history (current or former). A total of 336 (42%) patients had documented stage IV disease at the time of their initial diagnosis, and 321 (40%) had poorly differentiated tumors. The median follow-up time from initial diagnosis was 41 months. Median OS of the entire cohort of 803 patients at MD Anderson was 19 months (range 0.07-348).<br>For the PANCAN cohort (n=408), The median age at the time of diagnosis was 65 years (range 36-88). 46% were female and 54% were male. The median follow-up time from diagnosis was 15 months. Disease staging information was not available in majority of the patients in this cohort (59.8%). 23.8% (n=97) patients had documented stage IV disease at the time of diagnosis.
Recruitment	NA
Ethics oversight	The MD Anderson Cancer Center Institutional Review Board (IRB) approved the collection of demographics, clinical, and pathological information under IRB protocol 09-0373 and 2023-0091.

Note that full information on the approval of the study protocol must also be provided in the manuscript.

Field-specific reporting

Please select the one below that is the best fit for your research. If you are not sure, read the appropriate sections before making your selection.

☒ Life sciences ☐ Behavioural & social sciences ☐ Ecological, evolutionary & environmental sciences

For a reference copy of the document with all sections, see nature.com/documents/nr-reporting-summary-flat.pdf

Life sciences study design

All studies must disclose on these points even when the disclosure is negative.

Sample size	NA. Descriptive retrospective analysis.
Data exclusions	NA
Replication	External validation cohort from PANCAN
Randomization	NA
Blinding	NA

Reporting for specific materials, systems and methods

We require information from authors about some types of materials, experimental systems and methods used in many studies. Here, indicate whether each material, system or method listed is relevant to your study. If you are not sure if a list item applies to your research, read the appropriate section before selecting a response.

Materials & experimental systems

n/a	Involved in the study
☒	☐ Antibodies
☒	☐ Eukaryotic cell lines
☒	☐ Palaeontology and archaeology
☒	☐ Animals and other organisms
☐	☒ Clinical data
☒	☐ Dual use research of concern
☒	☐ Plants

Methods

n/a	Involved in the study
☒	☐ ChIP-seq
☒	☐ Flow cytometry
☒	☐ MRI-based neuroimaging

Clinical data

Policy information about clinical studies
All manuscripts should comply with the ICMJE guidelines for publication of clinical research and a completed CONSORT checklist must be included with all submissions.

Clinical trial registration

NA

Study protocol

NA

Data collection

The MD Anderson Cancer Center Institutional Review Board (IRB) approved the collection of demographics, clinical, and pathological information under IRB protocol 09-0373 and 2023-0091. Informed consent was waived, as per the IRB guidelines for retrospective studies of previously collected clinical and molecular information. The Palantir Foundry software system (Palantir, Denver, CO) was used to query the MD Anderson EHR to identify patients with a confirmed diagnosis of PDAC who underwent somatic tumor tissue mutation testing at MD Anderson from 3/14/1997 to 4/27/2023 for inclusion in the study.
Patient demographics, histopathology, tumor grade, surgical history, and mutational profiles were collected from the MD Anderson EHR and tumor registry data using the Foundry system. Histologic classification and grade were collected from the patients' pathology reports. Molecular testing was performed at MD Anderson's molecular diagnostics laboratory, which is College of American Pathologists (CAP) accredited and Clinical Laboratory Improvement Amendments (CLIA) certified. The gene panels used evolved during the study inclusion period, with expanding lists of genes over time. The information on tumor genomic alterations (GAs) was extracted from the available clinical and molecular data. Deidentified information was used for analysis.
For the validation PANCAN dataset, PanCAN, in partnership with Tempus (Tempus Labs Inc., Chicago, IL), offers the Know Your Tumor® (KYT) precision medicine service to patients with pancreatic cancer. KYT data is available through the PanCAN SPARK platform (www.pancan.org/spark). Tempus processes, sequences and conducts group-level bioinformatics analyses on tumor biopsy samples. Data is derived from the Tempus xT NGS panel that covers 648 genes with actionable oncologic mutations.

Outcomes

Overall survival (OS, from the time of initial diagnosis) was calculated from the date of initial diagnosis until death or last known contact.
